# The combined influence of body size and density on cohesive sediment resuspension by bioturbators

**DOI:** 10.1038/s41598-018-22190-3

**Published:** 2018-03-01

**Authors:** Francesco Cozzoli, Tjeerd J. Bouma, Pauline Ottolander, Maria Salvador Lluch, Tom Ysebaert, Peter M. J. Herman

**Affiliations:** 1 Department of Estuarine and Delta Systems, Royal Netherlands Institute of Sea Research (NIOZ) and Utrecht University, Yerseke, The Netherlands; 2 Institute for Marine Resources and Ecosystem Studies (IMARES), Wageningen University, Wageningen, The Netherlands; 3 Present Address: Dipartimento di Scienze e Tecnologie Biologiche ed Ambientali, Centro Ecotekne Pal. B S.P. 6, Lecce, Monteroni, Lecce Italy

## Abstract

We propose an empirical framework to scale the effects of bioturbation on sediment resuspension to population bioturbation activity, approximated as population metabolic rate. Individual metabolic rates have been estimated as functions of body size and extrapolated to population level. We used experimental flumes to test this approach across different types of marine, soft-sediment bioturbators. We observed that a large part of the variance in biota-mediated sediment resuspension can be explained by a positive relationship with population metabolic rate. Other mechanisms can strongly influence the outcome, such as bioturbation of deep sediment strata, biotic interactions with hydrodynamic stress and overlapping areas of influence must be further investigated. By relating the biota-mediated changes in resuspended sediment to metabolism, we can place our observations within the broader context of the metabolic theory of ecology and to formulate general expectations about changes in biota-mediated sediment resuspension in response to changes in population structure and climate change.

## Introduction

Macrobenthic infauna may act as ecosystem engineers^1,2^ by decreasing sediment stability and increasing sediment erodability with their bioturbating activities^3–5^. This reworking of sediment usually results from their feeding routines, either through directly swallowing the sediment to extract nutrients (*e*.*g*. *Arenicola marina*^6^) or perturbing the sediment surface while grazing (*e*.*g*. *Limecola balthica*^5^). Sediment stability may also be influenced by the respiration and filtration activity of filter feeders, such as the disturbances caused by valve abduction and deposition of pelleted pseudo-faeces (*e*.*g*. *Cerastoderma edule*^7^,). Finally, burrowing and moving activities are also likely to loosen the superficial sediment^8^. Recent studies showed that bioturbation activities are mostly effective in enhancing the resuspension of cohesive (muddy) sediment, whereas they do not affect the resuspension of not-cohesive (sandy) sediment^9^.

Although some types of deposit feeders are also able to trap the resuspended sediment by dampening hydrodynamic stress with their physical structure (*e*.*g*. tube-building worms like *Lanice conchilega*^10^,), the main mode of action for the majority of bioturbators is to render the sediment surface less resistant to erosion and to expose fine, easily erodible particles to the buoyant action of water^5^. Therefore, the sediment reworking rate by bioturbators (g m^−3^ time^−1^) can be related to the resuspension rate of the superficial sediment layer (g m^−2^ time^−1^). This in turn is related to change in the elevation of the sediment (cm time^−1^), which is an effective measure of change in habitat morphology. The effect of bioturbators on sediment resuspension can impact the overall development of coastal morphology^4,11^, and should be taken into account when forecasting the evolution of landscapes and ecosystems (e.g.^12,13^). Since bioturbators also oxygenate the sediment and enhance the transfer of particles and nutrients within the sediment layers and from the sediment surface to the water column^14^, they can strongly influence biogeochemical cycles^12,15–17^, species coexistence^18–20^ and aquatic food webs^21–23^.

Formulating a general quantitative framework to measure bioturbation processes is made difficult by the high diversity in macrozoobenthic species in terms of distribution, characteristics and functional behaviours^24–27^. In addition to characteristics of benthic populations such as overall biomass and density, previous approaches to this topic have used functional trait classification for bioturbators that requires taxonomic and life-history expertise, which are not always available (e.g.^5,12,13,27,28^). However, other empirical studies have pointed out that simple knowledge of an organism’s biovolume may allow for relatively simple estimates of the reworking intensity of surface sediment across a range of functional diversity^29^. To develop a general approach to scale the effects of bioturbation on sediment erodability across a broad range of functional groups, we tested the effect of a fundamental ecological descriptor, the metabolic rate.

### Theoretical framework

The positive scaling of bioturbation rates with metabolism can be assumed *via* the positive scaling of respiration, feeding and moving rates. An organism’s metabolic rate *I* (W) is^30,31^ expected to scale with individual size *M* (g), with an allometric exponent of ~0.75.

1 $$I \sim a{M}^{{0.75}}$$

where *a* is a normalization constant accounting for variability in metabolic rates independent from the effect of size (*e*.*g*. thermoregulatory strategy^32^). The positive size scaling of metabolic rates expressed from Equation 1 is considered to be one of the most fundamental patterns in ecology^33–38^, however, the actual values of the measured scaling exponents can exhibit large environmental^39,40^, taxonomic (*e*.*g*.^41–43^) and phenotypic (*e.g.*^44^) variability so that the “universality” of the ~0.75 scaling exponent is subject to ongoing debate. Supporting a metabolic approach, Gilbert *et al*^29^. showed that individual biovolume is a good predictor for sediment reworking intensity. Significant and positive power laws have been commonly observed between the size of macrozoobenthic organisms, their standard metabolic rate^45–47^ and other activities affecting stability, such as sediment ingestion/egestion (*e*.*g*.^48^ for *A*. *marina*^49^, for benthic detritivores^50^, for *C*. *edule*), burial and transport (*e*.*g*. bioturbation potential^12^, burying depth of *A*. *marina*,^48^; burying depth of *L*. *balthica*^51^). Ideally, if macrozoobenthos rework sediment proportionally to their individual energetic requirements, the amount of sediment loosened by a bioturbator and made available for resuspension (*R*, g) should change with the bioturbator size proportionally to the metabolic rate *I*:

2 $$R \sim I\,$$

The relationship between body size and density is an essential link between individual and population traits, such as population spatial density^34,37,52–54^. The overall energetic requirements of for a population of *N* (N of Ind. m^−2^) bioturbators of *M* size, *I*_*TOT*_ (W m^−2^), may be estimated as the product of the individual metabolic rates and the individual density^55^:

3 $${I}_{TOT} \sim NI$$

It follows that, at the variation of *I*_*TOT*_, the overall amount of resuspended sediment *R*_*TOT*_ (g m^−2^) may be approximated from the linear model

4 $${R}_{TOT}=c+d\ast {I}_{TOT}$$

where *c* is a normalization constant accounting for the amount of sediment that is resuspended due to physical forces only (i.e. in absence of bioturbation) and the coefficient *d* accounts for the relation between variation in *I*_*TOT*_ and variation in *R*_*TOT*_. The coefficient *c* is expected to be strongly dependent on the physical conditions under which the sediment resuspension happens^56,57^. The coefficient *d* may vary according to the modalities that different functional groups of bioturbators have in reworking sediment^58^.

### Experimental test

Using artificially created single-species or similar functional group assemblages allows us to isolate particular aspect of bioturbation, and thereby provide much needed mechanistic insight^13^. In this study, we test the hypothesis that the amount of resuspended sediment due to bioturbation action is proportional to the overall activity of the bioturbator population, expressed as a linear combination of individual metabolic rates and density of individuals. Thus, we used an experimental set-up that excluded variation in physical conditions (e.g sediment granulometry, cohesiveness, compaction, shear stress^57^,) or metabolic and behavioural changes in response to environmental cues (*e*.*g*. sediment composition,^9^; acidification,^39^; temperature,^59^; food availability^60^; shear stress^61^,). This simplification was made to test if bioturbation fits within the ecological framework of size-dependent energetic theories (*e*.*g*.^33^).

We tested our hypotheses using an empirical dataset. This dataset uses the mass of resuspended sediment (*MRS*, g m^−2^) as a measure of bioturbation effects on *R*_*TOT*_. The *MRS* in the water is coupled with the mass of bottom sediment by a dynamic balance between deposition and erosion. Increasing bottom shear stress has the effect to increase the sediment erosion and decrease the sediment deposition, thus increasing the *MRS*. By loosening the sediment with their activities, benthic bioturbators increase the amount of sediment available for resuspension at a certain current velocity, shifting the balance point between erosion and deposition to a higher *MRS*^5^. Analogously to previous studies (*e*.*g*.^3,5^) we did not measure sediment deposition and we only consider the effect of bioturbations on the equilibrium *MRS* (deposition rate = erosion rate) reached at a given level of bed shear stress from water motion.

The dataset encompasses a range of functional diversity (from shallow to deep bioturbators), individual densities (13 to 382 Ind. m^−2^) and body sizes (10 to 1136 mg Ash Free Dry Weight, AFDW). Individual metabolic rates were estimated according to the empirical model of Brey^62^. Three functionally different types of bioturbation activity were accounted for in the analysis^58^: (i) shallow-burrowing bivalves that make very shallow perturbations in the sediment by crawling on the surface, shaking valves and producing pelleted pseudo-faeces, represented by *C*. *edule*; (ii) Intermediate Burrowing Bivalves (IBBs) that disrupt the sediment surface by inhaling the sediment through their siphons and depositing pseudo-faeces, represented by *Abra alba*, *Scrobicularia plana*, *L*. *balthica* and *Ruditapes philippinarum*; and (iii) deep-burrowing Polychaeta that swallow surface sediment through a feeding funnel and expel it in the form of pseudo-faeces, forming characteristic feeding pits and pseudo-faeces casts, represented by *A*. *marina*. By using three contrasting functional groups with each group containing individuals that greatly vary in body size, we were able to measure the changes in sediment resuspension due to different modes of mechanical destabilisation across a large range of body sizes and densities.

## Results

The estimated individual metabolic rate of experimental organisms varied from 0.07 mW [+/−0.02 95 CI] (smallest size class of *C*. *edule*) to 2.16 mW [+/−0.49 95 CI] (largest size class of *A*. *marina*) (Table 1). The overall metabolic rate of the experimental populations (as a sum of the metabolic rates of homogeneously sized individuals of the same species) varied from 2.32 mW m^−2^ [+/−0.69 95 CI] (lowest density of *A*. *marina*) to 206.06 mW m^−2^ [+/−46.94 95 CI] (highest density of largest *A*. *marina*). Reported confidence intervals are representative of the cumulative effects on errors on the animal measurements, on the conversion between length or wet weight to AFDW and of the conversion between AFDW and metabolic rate.

**Table 1:** Table of treatments and results.

Species	Size	±95% CI	M	±95% CI	I	±95% CI	N	I_TOT_	±95% CI	R_TOT_	±95% CI
	mg/mm		mg AFDW		mW		N of Ind. m^−2^	mW m^−2^		g m^−2^	
*A*. *marina*	160	8	16.34	1.68	0.07	0.02	32	2.32	0.69	21.53	6.43
*A*. *marina*	160	8	16.34	1.68	0.07	0.02	64	4.64	1.38	38.58	2.43
*A*. *marina*	160	8	16.34	1.68	0.07	0.02	95	6.95	2.07	42.26	5.75
*A*. *marina*	160	8	16.34	1.68	0.07	0.02	127	9.27	2.77	39.41	0.84
*A*. *marina*	1500	75	169.04	20.57	0.51	0.13	32	16.11	4.02	35.64	4.86
*A*. *marina*	1500	75	169.04	20.57	0.51	0.13	64	32.22	8.04	41.43	2.13
*A*. *marina*	1500	75	169.04	20.57	0.51	0.13	95	48.33	12.07	48.88	6.7
*A*. *marina*	1500	75	169.04	20.57	0.51	0.13	127	64.44	16.09	60.88	23.3
*A*. *marina*	8000	200	970.27	148.4	2.16	0.49	32	68.69	15.65	45.51	2.86
*A*. *marina*	8000	200	970.27	148.4	2.16	0.49	64	137.37	31.29	42.89	3.36
*A*. *marina*	8000	200	970.27	148.4	2.16	0.49	95	206.06	46.94	145.29	100.92
*A*. *alba*	15	0.5	17.29	4.27	0.1	0.03	45	4.25	1.32	36.81	8.18
*A*. *alba*	15	0.5	17.29	4.27	0.1	0.03	95	9.1	2.82	37.29	0.31
*L*. *balthica*	15	0.5	33.98	4.57	0.16	0.04	33	5.02	1.12	34.24	2.92
*L*. *balthica*	15	0.5	33.98	4.57	0.16	0.04	64	10.03	2.24	36.13	5.17
*L*. *balthica*	15	0.5	33.98	4.57	0.16	0.04	191	30.09	6.71	41.05	0.23
*L*. *balthica*	15	0.5	33.98	4.57	0.16	0.04	382	60.19	13.43	49.67	7.66
*S*. *plana*	15	0.5	17.98	2.61	0.1	0.02	64	6.24	1.46	44.96	9.22
*S*. *plana*	15	0.5	17.98	2.61	0.1	0.02	382	37.46	8.76	88.84	87.85
*S*. *plana*	35	0.5	166.26	17.99	0.51	0.1	64	32.74	6.46	63.32	40.89
*R*. *philippinarum*	25	0.5	159.54	46.73	0.5	0.17	32	15.87	5.33	34	14.53
*C*. *edule*	10	0.5	10.52	2.42	0.02	0.03	95	6.28	7.89	37.26	7.89
*C*. *edule*	10	0.5	10.52	2.42	0.02	0.03	191	12.57	1.15	43.63	1.15
*C*. *edule*	10	0.5	10.52	2.42	0.02	0.03	382	25.13	10.34	59.09	10.34
*C*. *edule*	20	0.5	99.14	12.52	0.07	0.1	32	11.14	9.01	44.04	9.01
*C*. *edule*	20	0.5	99.14	12.52	0.07	0.1	64	22.27	0.4	44.65	0.4
*C*. *edule*	20	0.5	99.14	12.52	0.07	0.1	127	44.55	2.23	45.04	2.23
*C*. *edule*	20	0.5	99.14	12.52	0.07	0.1	255	89.09	34.37	71.37	34.37
*C*. *edule*	35	0.5	606.31	70.85	0.27	0.31	13	17.17	0.11	36.61	0.11
*C*. *edule*	35	0.5	606.31	70.85	0.27	0.31	32	42.92	11.72	48.95	11.72
*C*. *edule*	35	0.5	606.31	70.85	0.27	0.31	64	85.84	6.63	49.55	6.63
*C*. *edule*	35	0.5	606.31	70.85	0.27	0.31	191	257.51	20.26	122.62	20.26

Specimens are representative of deep (*A*. *marina*) intermediate (*A*. *alba*, *L*. *balthica*, *S*. *plana*, *R*. *philippinarum*) and shallow (*C*. *edule*) bioturbation modalities. Homogeneously size individuals were selected according to their shell length (mm. bivalves) or wet weight (mg. *A*. *marina*). Individual size was converted in individual body mass (M. mg AFDW) according to the empirical relationship provided from the NIOZ – Yerseke Monitor Taskforce. The individual metabolic rate (I. mW) was estimated according to the empirical relationships provided from Brey^62^. The population metabolic rate (I_TOT_. mW m^−2^) was estimated as the product between the individual rates I (mW) and population density N (N. of Ind m^−2^). 95% CIs reported in table accounts from error propagation across the different conversion steps. The observed mass of resuspended sediment (R_TOT_. average of two replicates) is reported in g m^−2^.

**Figure 1:**
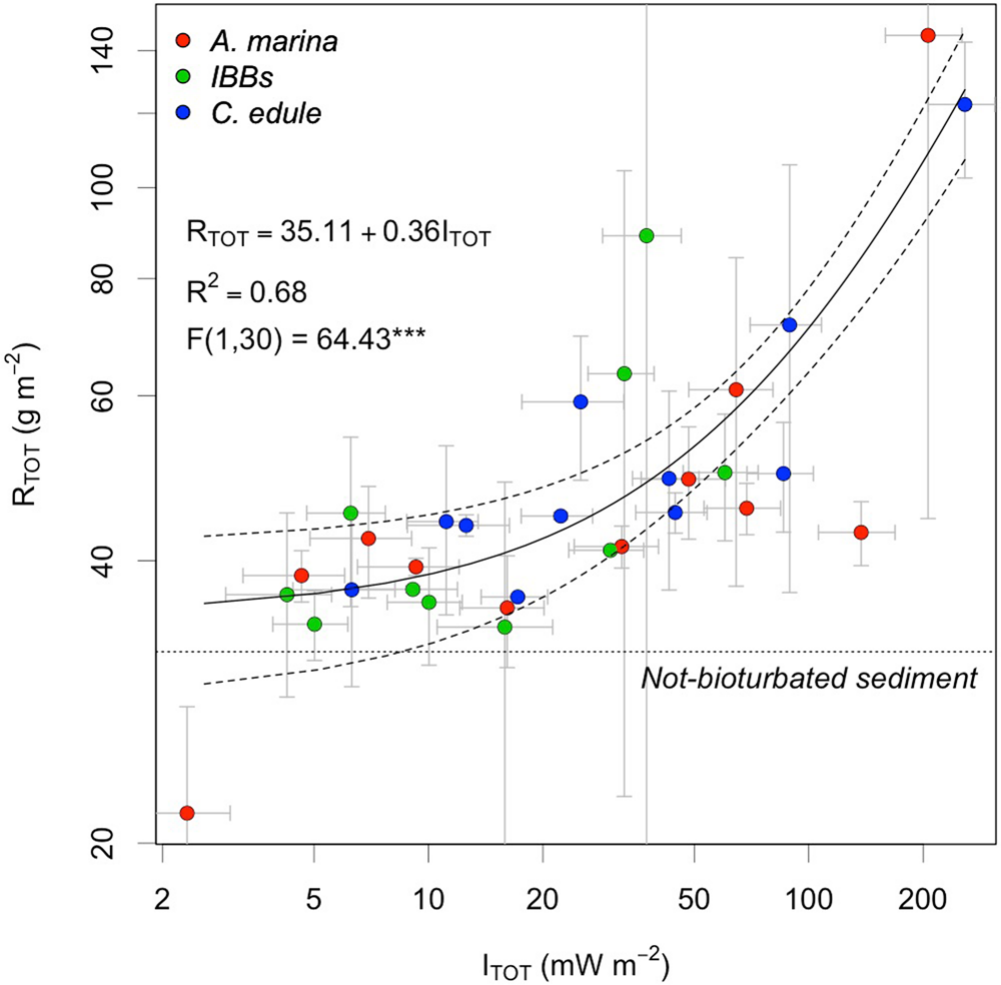
Mass of resuspended sediment (*R*_*TOT*_. g m^−2^) *vs*. bioturbators population metabolic rate (*I*_*TOT*_. mW m^−2^). Measures were obtained from deep (*A*. *marina*. red) intermediate (Intermediate Burrowing Bivalves, green) and shallow (*C*. *edule*, blue) bioturbators. Error bars show the standard errors on both *R*_*TOT*_ (variability between the two replicates) and *I*_*TOT*_ (approximation from bioturbators size measurements and metabolic rate estimation). The full line shows the estimated linear relationship. Dashed lines show the 95% CI.

On average, 32.25 g m^−2^ [+/−1.73 95 CI] of sediment was resuspended in the control runs without bioturbators at a bed shear stress of 0.18 Pa. When the same shear stress was applied after 48 h of active bioturbation in the flumes, we observed a major difference in sediment resuspension: *R*_*TOT*_ increased up to almost 5 times in the bioturbated runs than in the non-bioturbated controls. The highest *R*_*TOT*_ (145.29 g m^−2^ [+/−100.92 95 CI]) was observed during activity by the highest density of the largest *A*. *marina*. Only in one case (the lowest density of the smallest *A*. *marina*) did we observe a lower amount of resuspended sediment than in the defanauted control (Fig. 1).

Spearman’s rank correlation between overall population metabolism and *R*_*TOT*_ is 0.82 (p-value < 0.001), consistently exceeding the corresponding correlations with individuals’ density only (0.37, p-value < 0.05). A large part of the observed variation in *R*_*TOT*_ (R^2^ = 0.67) could be described as a linear function of the overall population metabolism:

5 $${R}_{TOT}=35.11+0.36\ast {I}_{TOT}$$

The estimated intercept (35.11 g m^−2^ [+/−6.60 95 CI]) is consistent with the resuspended sediment observed in the defanauted control. Stepwise-model simplification showed that differences across functional groups were not statistically significant (p-value > 0.1, Table 2).The trend is still significant (p-value < 0.05) and positive (scaling coefficient 0.17 [+/−0.15]) when the two observations with higher *I*_*TOT*_ are removed. (Appendix C, Table C3). We did not detect linear correlation between the amount of sediment resuspended per unit of metabolic power *R*_*BIO*_ = *(R*_*TOT*_ − *R*_*CONTROL*_*)/I*_*TOT*_ (g mW^−1^) and both bioturbator density and individual size (Table 3). Independently from the size-density ratio of the experimental population, the *MRS* increases on average 0.47 g [+/−0.37 95 CI] for each mW of population metabolic power, a value consistent with the estimate of the coefficient *d* in Equation 5 (0.36 [+/−0.08 95 CI]).

**Table 2:** Summary of the regression models between *R*_*TOT*_ (g m^−2^) and bioturbators population metabolic power (*I*_*TOT*_. mW m^−2^) for different functional groups (FunGroup) of bioturbators: deep (*A*. *marina*) intermediate (*IBBs*) and shallow (*C*. *edule*). Steps of explanatory variables selection (interactive effect of the categorical variable. cumulative effect of the categorical variable. excluding the categorical variable) are shown including their AIC values. The selected model (excluding the categorical variable) is marked in bold.

	Response								
	R_TOT_ ~ FunGroup * I_TOT_			R_TOT_ ~ FunGroup + I_TOT_			R_TOT_ ~ I_TOT_		
	Est.	95% CI	p	Est.	95% CI	p	Est.	95% CI	p
*c*	28.28	16.09–40.46	<0.001	31.01	20.61–41.42	<0.001	**35.11**	**28.51–41.71**	<**0.001**
IBBs	7.10	−12.23–26.43	0.457	7.80	−5.65–21.24	0.245			
C. edule	8.56	−8.36–25.48	0.308	3.07	−9.68–15.83	0.625			
I_TOT_	0.42	0.27–0.57	<0.001	0.37	0.28–0.47	<0.001	**0.36**	**0.27–0.45**	<**0.001**
IBBs:I_TOT_	0.11	−0.45–0.68	0.688						
C. edule:I_TOT_	−0.10	−0.30–0.10	0.312						
Observations	32			32			**32**		
R^2^/adj. R^2^	0.714/0.658			0.698/0.665			**0.682/0.672**		
F-statistics	12.954***			21.530***			64.427***		
AIC	270.397			268.133			**265.712**

**Table 3:** Summary of the regression models between the amount of resuspended sediment per unit of population metabolic power (*R*_*BIO*._ g mW^−1^) and bioturbators density (N of Ind. m^−2^) and individual size (mg AFDW) Steps of explanatory variables selection are shown including their AIC values. The selected model (excluding the categorical variable) is marked in bold.

	*Response*								
	R_BIO_ ~ Density * Size			R_BIO_ ~ Density + Size			**R**_**BIO**_		
	Est.	95% CI	p	Est.	95% CI	p	**Est**.	**95% CI**	**p**
Intercept	0.26	−0.47–0.99	0.477	0.28	−0.42–0.97	0.417	**0.47**	**0.09–0.84**	**0.017**
Density	0.00	−0.00–0.01	0.305	0.00	−0.00–0.01	0.305			
Size	0.00	−0.00–0.00	0.952	−0.00	−0.00–0.00	0.805			
Density:Size	−0.00	−0.00–0.00	0.794						
Observations	32			32			**32**		
R^2^/adj. R^2^	0.048/−0.054			0.046/−0.020			**0.000/0.000**		
AIC	100.882			98.961			**96.459**		

## Discussion

In this paper, we derived relationships across species to describe the potential effect of bioturbators on the amount of sediment made available for resuspension. We hypothesised that the increase *per area* of resuspended sediment due to mechanical destabilisation by bioturbation is proportional to the overall activity by the bioturbator population, so that it can be quantified as a positive function of individual metabolism and density of individuals. We were able to test these hypotheses by measuring the effect of different functional groups of bioturbators on sediment erodability. Confirming our expectations, the positive scaling with population metabolic rate was able to explain a large portion of the variance (67%) in loosened and resuspended sediment due to bioturbation. The scaling relationship was not significantly different among the investigated groups, supporting the idea of common energetic constraints acting across a large range of functional diversity (Fig. 1, Table 2).

Equation 5 provides an empiric description of bioturbation effects on sediment resuspension. However, the expected link may not be mechanistically transferable from the individual to the population level purely on the basis of body size-density relationships. As an example, individuals may have overlapping areas of influence, that vary with organisms’ density^3,5^ and size^63^. We observed a constant amount of resuspended sediment per unit of metabolic power (*R*_*BIO*_) across density and size gradients (Table 3), indicating that negative interference was not apparent in the data. However, it must be considered that such interference may occur at the increase of both the individuals’ size and density. As an example, at *L*. *balthica* densities (500, 1000, 1500 Ind m^−2^) higher than those we tested in our experiment (maximal tested density 382 Ind m^−2^), the per capita amount of bioturbated sediment decreases with increasing density^3^. It follows that a limiting function should be used to apply Equation 5 to very high individuals densities (*e*.*g*. >500 Ind m^−2^ for *L*. *balthica*^3,5^,). As an example, Willows *et al*.^3^ used an asymptotic function to model the relationship between *L*. *balthica* density and resuspended sediment, and van Prooijen *et al*.^5^ used an exponential function to model the probability of overlap in influence area. However, such high densities of bioturbators are not very likely to be realized in nature, *e*.*g*. *L*. *balthica* has an individual density lower than the maximal one we tested, 382 Ind m^−2^, in the 97% of the records collected between 2005 and 2011 in the Westerschelde and Oosterschelde,^64^. Thus, the use of a limiting function for individuals’ density may eventually be neglected for broad field applications of Equation 5.

Our measures, similarly to those of previous studies^3,5^ focus on sediment resuspension and neglect effects on deposition that may be relevant at high individuals density or at the decrease of the bed shear stress. Because bioturbators can increase the sediment surface roughness with their physical shape (i.e. protruding shells of *C*. *edule*) or with the structures they produce (i.e. feeding funnels of Intermediate Burrowing Bivalves (IBBs), pseudo-faeces casts of *A*. *marina*), high densities of bioturbators could dampen the near-bottom water flux and reduce the bed shear stress, thereby trapping the sediment in saltation before resuspension. The reworking of the sediment by the animals could also change the characteristics of the sediment: larger flocs can be broken, or excreted sediments can be pelletized and compacted. The finest part of the sediment, exposed to the buoyant action of water from sediment mixing, may be eroded at first, increasing the average granulometry and decreasing the cohesiveness of the bottom sediment^65^. It is possible that these processes may change the sediment response to the water action, inducing changes in the equilibrium *MRS*. Finally, it is also possible that individuals could decrease their metabolic/activity rates with increasing density (*e*.*g*.^66,67^), generating negative covariance between *I* and *N*.

The aforementioned sources of negative correlations between bioturbators size, density and metabolic rate may generate non linearites in the relationship between *R*_*TOT*_ and *I*_*TOT*_. At the scale of our analysis, these non-linearities are intrinsically included in Equation 5 and may result in a smaller scaling coefficient *c*. Detailed measurements are required to a deeper understanding of these interactions. It must be considered that our model has also a substantial prediction error and might be affected from the two observations with higher overall metabolic rates (largest sizes and densities of *A*. *marina* and *C*. *edule*, Fig. 1). This indicates that the experimental design could be improved by increasing replications and distributing experimental effort more evenly across the range of overall population metabolism. However, excluding the two observations with higher leverage the scaling trend is still positive and significant (p-value < 0.05 Appenidx C, Table C3). The logistic efforts necessary for empirical testing (in our case, each single run took ca. 1 week of preparation followed by 1 week of experiments) it is the main reasons that our dataset is limited. While the dataset we collected may be regarded as not being “optimal”, it is one of the most complete experimental datasets (to our knowledge) on biota-mediated sediment resuspension that has been measured according to metabolic and density gradients across different species.

### Comparison with previous approaches

Positive values for size and density scaling have previously been proposed as descriptors of bioturbation potential. For example, the bioturbation potential index (*BP*_*c*_^12,24,27^,) for marine ecosystems approximates the effects of benthic bioturbators as a linear combination of abundance, average size and behavioural traits. Applications of *BP*_*c*_ showed that benthic community structure can be actually used to predict the process of bioturbation in real ecosystems^13^. However, this approach has some limitations. First, it requires extensive knowledge of species life histories and high taxonomic expertise. In particular, the *BP*_*c*_ index relies on detailed functional classification of organism traits associated with sediment mixing and motility^27^. The paucity of such information for the majority of marine species is a source of concern, as this information is needed to project potential changes in *BP*_*c*_ under future policy or environmental scenarios^27^. Second, combining individual size, density and functional traits is potentially redundant in the sense that different functional groups and motility classes may already contain relationships combining body size and size/density ratios^68^. Third, the *BP*_*c*_ index, being based on a linear combination of abundance, biomass and behavioural traits, represents changes in bioturbation potential as a function of changes in species composition (which is difficult to predict in any detail) and in the biomass/abundance ratio on which typical body size is calculated^27^. Our experiment showed that different functional behaviours and motility classes did not have significant influence the amount of sediment resupended due to bioturbation. The largest part of observed variance in resuspended sediment could be explained simply in terms of bioturbators’ population metabolic rate. Hence, a coarser and less taxonomically demanding classification of bioturbators than used for the *BP*_*c*_ index would still able to predict the effect of bioturbation processes on sediment resuspension. This is supported by other studies showing that indicators based on community size structure, rather than on species-specific characteristics, can be used for describing the ecosystem status for some functional aspects^69^.

### Scope for extrapolation

Sediment resuspension can originate from very diverse interactions and activities^4^, which may also depend on external conditions. While our measures allow for general quantifications of scaling effects across a range of bioturbator diversity, other issues must be investigated for applications to field contexts. Seawater temperature is a key regulator of metabolic rates in ectotherms, such as macrofaunal invertebrates^70^. Individual metabolic rates are expected to increase with higher temperature within the ranges of physiological thermal tolerance^32,59,70–74^. From this perspective, investigations into the effects of temperature on bioturbation rates are relevant for estimating the potential effect of seasonal, latitudinal and global climate change on biota-mediated sediment resuspension. Increases in bioturbation activity with increasing temperature have been observed (*e*.*g*.^75–77^), supporting the hypothesis that there is a metabolic dependence of large-scale bioturbation effects. Although our experiments were performed at a fixed temperature, the amount of resuspended sediment was related to the population metabolism than to density, biomass or biovolume (*e*.*g*.^29^). Having such a metabolism-based relationship does enable speculative extrapolations on how predicted temperature increases may influence bioturbation effects.

The temperature dependence of metabolic rates can be described according to the Boltzmann-Arrhenius exponential model *e*^*−E/kT*^, where *k* is Boltzmann’s constant, *E* is the mean activation energy of metabolic reactions and *T* is the Kelvin temperature^32,59,70–74^. Assuming that all other mechanisms involved in sediment resuspension do not change with temperature variations, expectations about the effect of increasing temperature on *R*_*TOT*_ *via* metabolic rates can be formulated including a Boltzmann-Arrhenius temperature scaling^59^ in the metabolic term *I* of Equation 3:

6 $${R}_{TOT}=c+d\ast N(a{M}^{0.75}{e}^{-E/kT})$$

Regarding seasonality, our experimental temperature (18 °C) is representative of average water temperature of the Westerschelde in August/September, warmest period of the year. The average water temperature in February/March, coldest period of the year, is around 7.2 °C. According to Equation 6, the winter temperature should generate a decrease of 63% in metabolic rates for the same bioturbators population and thus a proportional decrease in biota-mediated sediment resuspension with respect to what we measured.

Previous studies emphasised that the loss of large-sized species related to global warming could drastically reduce the potential for deep bioturbation, with negative consequences for global biogeochemical cycles^12,16^. In contrast, our observations suggest that an increase in individual metabolic rates should actually lead to an increase in the amount of sediment made available for resuspension. In particular, according to Equation 6 an increase in summer temperature of 3 °C (as it is expected to happen in the southern part of the North Sea before the end of this century^78^,) should imply an increase in metabolic rates of 30% with respect to our reference temperature. Assuming that increases in individual metabolism will not be energetically balanced by a reduction in population density^40^, the positive scaling of metabolism with temperature would imply a consistent increase in bioturbation and biota-mediated sediment resuspension due to global warming at the end of this century.

Our estimations of changes in metabolic/bioturbation activity according to temperature are in the range of published empirical observations of variation in bioturbation activities^75–77,79^ with respect to temperature change. For practical applications, population metabolic rates and their effect on sediment resuspension can be estimated with good approximation from survey or predicted data on benthic community composition by using empirical models (*e*.*g*.^62^) or from theoretical expectations on size scaling of community metabolic rates (*e*.*g*.^33^). Together with fluctuations in population structure and size density ratio^80,81^, resource availability and behavioural adaptations^82^, scaling relationships between temperature, metabolism and *R*_*TOT*_ may contribute to explain and model the temporal^83^ and spatial^84^ variation observed in field bioturbation activity and contribution to sediment resuspension. Other parameters influencing aquatic invertebrates metabolism and energy allocation (*e*.*g*. depth, sex, age^44,62^) may be included to adapt the metabolic rate according to the environmental conditions and physiological status of the bioturbating population to which Equation 5 is applied.

Beyond their effect on metabolic rates, the influence of local environmental conditions (*e*.*g*. sediment granulometry, current velocity, anthropogenic disturbance) should be assessed because this can affect bioturbator distribution (*e*.*g*.^85–87^), as well as their interaction with the physical factors involved in sediment resuspension (*e*.*g*. shear stress^61^, temperature and acidification^39^, sediment composition ^9^, and wave exposure^88^). These factors can strongly influence the outcome of bio-mediated sediment resuspension. It is straightforward that different combinations of environment physical properties as sediment properties and bed shear stress will result in a different coefficient for physical erosion *c* in Equation 4^57^. Also the coefficient *d* relating population metabolism and sediment resuspension may vary as a function of environmental conditions. For example, it is possible that at values of bed shear stress higher than those tested in our experiment (i.e. allowing the water current to erode the deeper sediment strata and to completely smooth out all the roughness of the sediment surface generated by high densities of bioturbators) may result in steeper relationships being observed. However, such an increase in water energy may lead to physical factors and unpredictable erosion patterns (scouring) overcoming the importance of biological factors in determining sediment resuspension. Also, (i) intense shear stress is generally associated with non–cohesive sediment, where bioturbators have no or limited effect on sediment resuspension^9^ and (ii) high densities and large sizes of bioturbators are generally associated with low shear stress in nature^64,86^, which makes the combination of strong shear stress, cohesive sediment, high bioturbator density and large bioturbator size unrealistic.

Finally, our measurements were focused on single species and sizes classes in order to emphasize scaling relationships. However, the effects of individual species on sediment resuspension in a mixed benthic community may be complex, depending on how interspecific interactions affect the activity of the involved species; these must also be accounted for in order to extrapolate mesocosm observations to field contexts^11,89^. For example, positive correlations have been observed between benthic diatom abundance and both *C*. *edule*^90^ and *A*. *marina*^91^ biomass. Benthic diatoms are well known sediment stabilisers, able to glue together sediment grains by producing extracellular polymeric substance^92^ and to increase sediment resistance to erosion^93,94^. By disrupting and grazing the diatom film, benthic bioturbators may have a much higher relative impact on mudflat morphology than what we measured in our flumes because they are able to trigger the resuspension of sediment that is otherwise stabilised by diatoms^95^.

## Conclusions

Empirical descriptions of the behaviour of organisms are needed for integrated modelling of bio-mediated physical processes^5,96^. With this study, we developed a general approach to scale the effects of bioturbation on sediment erodability across a broad range of functional groups. By following the general predictions from body size effects on the energy expenditure of individual activities^33,62,97^, and scaling up the energetic budget from the individual to population level^55^, we showed that the effect of bioturbators on cohesive sediment resuspension can be described simply in terms of bioturbators’ population metabolic rate. While our quantitative estimation of increasing *R*_*TOT*_ with community metabolism must be treated with caution due to the relatively limited extent of our dataset and because we did not directly test the effect of temperature or other influential factors, it can still be indicative of trends in ecosystem functioning. Being based on such a highly fundamental ecological descriptor as metabolic rate, our observations can be placed within the framework of general ecological allometric theories and in particular to the Metabolic Theory of Ecology^33^, allowing to formulate general expectations about present and future trend in biotic contribution to sediment resuspension based on expected variations in community composition (*e*.*g*.^80,81,98^).

## Material and Methods

### Target organisms

The ecosystem engineers used for this experiment were the bivalves *Cerastoderma edule*, *Limecola balthica*, *Abra alba*, *Scrobicularia plana* and *Ruditapes philippinarum*, and the Polychaeta *Arenicola marina*. These organisms share a common habitat (mainly muddy intertidal flats), but they live in the sediment at different depths: from very shallow (*C*. *edule*, shells usually emerge from the sediment surface) to intermediate depths of 3 to 10 cm (*L*. *balthica*, *A*. *alba*, *S*. *plana* and *R*. *philippinarum;* grouped together as Intermediate Burrowing Bivalves or IBBs) to relatively deep depths below 10 cm (*A*. *marina*)^25,26^. Accordingly, their feeding modes vary from obligate suspension feeding (*C*. *edule*) to a mixture of suspension and deposit feeding (IBBs) to obligate deposit feeding (*A*. *marina*)^25,26^. While having similar individual energetic requirements^62^, obliged suspension feeders have been observed to have a lower size density ratio with respect to obligate deposit feeders, implying a different resource availability for the two guilds^68^.

The selected species are representative of three qualitatively different types of bioturbation activity^58^. *C*. *edule* reworking of sediment is mostly related to bio-deposition, vertical and horizontal movements and valve adduction^99^. *L*. *balthica*, *A*. *alba*, *S*. *plana* and *R*. *philippinarum* all burrow within ca. 5 cm from the sediment surface, and they can disrupt the sediment surface by inhaling the sediment with their siphons to graze on benthic diatoms^63^. *A*. *marina* swallow surface sediment through a feeding funnel and expel it in the form of pseudo-faeces, forming characteristic feeding pits and pseudo-faeces casts^6^. Given their similar function with respect to their sediment reworking modality, which sets them clearly apart from both *A*. *marina* and *C*. *edule*, we grouped these species into a single homogeneous group, i.e. IBBs. We also used this pooled approach to generate enough variation in body size and density for the intermediate burrowing, as this could not be realized at the species level in contrast to *A*. *marina* and *C*. *edule*. Not enough data were available to apply a robust statistical test to assess for inter-specific differences between size and density scaling within the IBB group. However, we did not detect any significant inter-specific differences when comparing the amount of suspended sediment observed from experiments using similar densities and sizes of the four species included in this group (see Appendix C, Tables C1 and C2). In addition, our direct observations and those of many other authors collected over the years (*e*.*g*.^27,58,63,100,101^) indicate that these organisms share common lifestyles, and modes of feeding and mobility. Finally, three of these species (*A*. *alba*, *L*. *balthica* and *S*. *plana*) belong to the same suborder (Tellinacea), two of them (*A*. *alba* and *S*. *plana*) to the same family (Semelidae), and they possess many physiological and structural similarities^102^.

The tested combinations of bioturbators’s body sizes and densities were selected in a way to cover the natural range of each analysed species (*e*.*g*.^6,25,26,48^) and according to the availability of experimental organisms (Table 1). Bioturbators’ mass (mg AFDW) was estimated from the specimens length (mm, bivalves) or wet weight (mg, *A*. *marina*) according to the relationships provided from the NIOZ – Yerseke Monitor Taskforce. Bioturbators’ individual metabolic rates were estimated according to the empirical model for acquatic macroinvertebrates respiration of Brey^62^ using a trait classification for sessile intertidal satiate Anellida and Bivalvia Heterodonta operating at 18 °C and assuming an average energy density of 21.5 J mg^−1^^103^. See Appendix A for more details about specimens’ measurements and calculation of metabolic rates.

Considering the large total number of flume runs needed (32 different combinations of size, density and functional groups × 2 replicates × 3 runs for combination = 192 runs, Table 1) and the time-consuming character of each flume experiment, the animals were collected between May 2011 and May 2012 from the intertidal flats of the Oosterschelde and Westerschelde. At the time of collection, average daily water temperature was between 14 and 17 °C. After collection, the animals were always allowed to acclimate for 1 week in containers filled with sediment and aerated filtered marine water that was kept at 18 °C (water temperature in the Westerschelde during full summer). Considering the relatively limited difference in temperature between field and mesocosms, one week of acclimation (rather than the two weeks usually adopted in macrozoobenthos studies) should be sufficient to reduce the risk of temperature shocks that could severely affect bioturbator metabolic rates. However, it is still possible that small deviations in bioturbator basal metabolic rates due on partial acclimation may have induced some minor bias in our estimates. Experiments were performed directly after this week of acclimation. Each flume run always used homogeneously sized individuals of a single species. For each species, different densities of individuals were tested in separate runs.

### Experimental equipment

The annular flumes we used are a variation of the design described by Widdows *et al*.^61^ (Appendix B, Figures B1–B3). The annular channel has a surface of 0.157 m^−2^. In the majority of the cases, we used flumes with an overall height of 40 cm. A modified version with an overall height of 80 cm was used to have a higher sediment column and allow the largest sized *A*. *marina* to settle properly. To avoid abiotic variability in resuspended sediment due to different sediment characteristics^94^ in the experiments, homogeneous, wet, muddy sediment (median grain size = 100 μm, silt content 12%) was put in a flume, mixed to a smooth mass and allowed to consolidate until creating a layer of ca. 10 cm height in the shorter flumes and of ca. 50 cm in the taller ones. Excess water in the sediment was drained through a pebbled bed placed at the bottom of the flume. After 48 hours, the flumes were filled with 31.4 L of filtered seawater (height of the water column 20 cm). To prevent damaging the sediment surface, a sheet of bubble plastic was placed on top of it before gently spraying water into the flume. A water current of 30 cm sec^−1^ was applied, corresponding to a bed shear stress of 0.18 Pa, which should be sufficient to resuspend the bioturbated sediment^61^. To apply the current, we used a smooth, adjustable rotating disk, which was driven by a microprocessor-controlled engine. An acoustic Doppler velocimetry probe was used to calibrate water velocity as a function of engine rotation speed. Water turbidity, as a proxy of resuspended sediment, was measured using an optical backscatter sensor (OBS 3+, Campbell scientific) facing the water perpendicularly to the current direction at 10 cm from the sediment surface, and converted into suspended sediment concentration (g L^−1^) based on calibration by gravitometric analysis (Appendix B, Table B1). To express sediment resuspension in spatial units, we converted the suspended sediment concentration to Mass of Resuspended Sediment (*MRS*, g m^−2^). Previous studies^5,61^ have shown that in cohesive sediment, mostly supply-limited erosion occurs, i.e. after the water motion has started, the *MRS* reaches equilibrium due to limitation of erodible material^56^. In our experiments, the equilibrium *MRS* was usually reached after ca. 5 minutes of applying current.

### Experimental protocol

Every experiment (2 replicates) included a preliminary run, a control run without added animals and an experimental run with benthic animals. All runs lasted 20 min and were repeated at intervals of 48 h. The aim of the first, preliminary run was to further smoothen and homogenise the sediment surface of the flume bottom. As a consequence of the limited erosion that occurred during this control run, a uniform, less than 0.5 mm-thick layer of fine sediment was deposited on the sediment surface of each flume within a few hours from the end of the run. Pilot experiments conducted in flumes without fauna, involving several sequential daily runs, showed some small differences across flumes but no increase in sediment resuspension compared to the control run on the first day. This implies that all the sediment available for resuspension in the absence of bioturbation had been eroded during the first run and was suspended again during the subsequent runs. This observation allows us (i) to use the second run without fauna as an independent measure for the amount of sediment resuspended due only to shear stress and (ii) to subsequently use this measure as an internal control to quantify the bioturbation effect, while minimizing the small differences across flumes.

Immediately after the control run, animals were introduced into the flumes and evenly distributed over the sediment surface. The aim of the third, experimental run was to measure the change in sediment resuspension with respect to the abiotic control resulting from the action of the bioturbators during the 48 hours in which they remained in the flumes. The choice of a longer time interval (48 h) compared with the typical interval between erosion stress peaks (typically 12 or 24 h in a tidal system) was necessary to give to the animals a chance to properly settle in the new environment and recover from manipulation stress. The vast majority of them were buried within a few minutes after being placed in the flume. *A*. *marina* generally did not move from the initial settlement point and produced a single feeding pit with a pseudo-faeces cast for each individual. Some individuals of *C*. *edule* and IBBs crawled on and below the sediment surface, leaving evident tracks.

### Data analysis

We tested the hypothesis that the effect of bioturbators on sediment resuspension (approximated as equilibrium Mass of Resuspended Sediment, g m^−2^ at a current shear stress of 0.18 Pa) is proportional to the overall population metabolic activity (*I*_*TOT*_, mW m^−2^, calculated as the product of the individual metabolic rate *I*, mW, and the population density *N*, N of Ind. m^−2^). To assess for differences in intercepts and scaling coefficients across the three groups of bioturbators, multivariate linear regression models were fitted with full interaction terms, including the dummy predictive variable “Functional Group”. Selection of predictive variables and interaction terms was assessed by a bi-directional elimination stepwise procedure.

The correlation between individuals’ size, density and amount of resuspended sediment per unit of metabolic power, *R*_*BIO*_ = *(R*_*TOT*_ − *R*_*CONTROL*_*)/I*_*TOT*_, g mW^−1^, was investigated to asses for interferences between individuals at increasing individuals’ density and size. Also in this case, selection of predictive variables and interaction terms was assessed by a bi-directional elimination stepwise procedure.

All analyses were performed within the free software environment R 3.3.2^104^.

## Electronic supplementary material

Supplementary information

## References

[1] Jones CG, Lawton JH, Shachak M (1994). Organisms as ecosystem engineers. Oikos.

[2] Jones CG, Lawton JH, Shachak M (1997). Positive and negative effects of organisms as physical ecosystem engineers. Ecology.

[3] Willows R, Widdows J, Wood R (1998). Influence of an infaunal bivalve on the erosion of an intertidal cohesive sediment: A flume and modeling study. Limnol. Oceanogr..

[4] Le Hir P, Monbet Y, Orvain F (2007). Sediment erodability in sediment transport modelling: Can we account for biota effects?. Conti. Shelf Res.

[5] van Prooijen BC, Montserrat F, Herman PMJ (2011). A process-based model for erosion of Macoma balthica-affected mud beds. Cont. Shelf Res..

[6] Zebe E, Schiedek D (1996). The lugworm Arenicola marina: A model of physiological adaptation to life in intertidal sediments. Helgolander Meersun.

[7] Ciutat A, Widdows J, Pope N (2007). Effect of Cerastoderma edule density on near-bed hydrodynamics and stability of cohesive muddy sediment. J. Exp. Mar. Biol. Ecol..

[8] Friedrichs M, Leipe T, Peine F, Graf G (2009). Impact of macrozoobenthic structures on near-bed sediment fluxes. J. Mar. Sys.

[9] Li B, Cozzoli F, Soissons LM, Bouma TJ, Chen L (2017). Bioturbation effect on the erodibility of cohesive versus non-cohesive sediments along a current velocity gradient: a case study on cockles. J. Exp. Mar. Biol. Ecol..

[10] Schonke M (2017). Impact of Lanice conchilega on seafloor microtopography off the island of Sylt (German Bight, SE North Sea). Geo Mar. Lett.

[11] Orvain F, Le Hir P, Sauriau PG, Lefebvre S (2012). Modelling the effects of macrofauna on sediment transport and bed elevation: Application over a cross-shore mudflat profile and model validation. Estuar. Coas. Shelf S.

[12] Solan M (2004). Extinction and ecosystem function in the marine benthos. Science.

[13] Queirós A (2015). Can benthic community structure be used to predict the process of bioturbation in real ecosystems?. Prog. Oceanogr..

[14] Ubertini, M., Lefebvre, S., Gangnery, A., Grangere, K., Le Gendre, R. & Orvain, F. Spatial variability of benthic-pelagic coupling in an estuary ecosystem: Consequences for microphytobenthos resuspension phenomenon. *Plos One* **7** (2012). 10.1371/journal.pone.0044155 PMC3430628 22952910

[15] Quintana, C. O. *et al*. Carbon mineralization pathways and bioturbation in coastal Brazilian sediments. *Sci*. *Rep*. (5) (2015). 10.1038/srep16122 PMC4630785 26525137

[16] Thomsen, M. *et al*. Consequences of biodiversity loss diverge from expectation due to post-extinction compensatory responses. *Sci*. *Rep*. **7** (2017). 10.1038/srep43695 PMC5334654 28255165

[17] Zhang, L. *et al*. The impact of deep-tier burrow systems in sediment mixing and ecosystem engineering in early Cambrian carbonate settings. *Sci*. *Rep*. **7** (2017). 10.1038/srep45773 PMC5379565 28374857

[18] Mermillod-Blondin F, Lemoine DG (2010). Ecosystem engineering by tubificid worms stimulates macrophyte growth in poorly oxygenated wetland sediments. Funct. Ecol.

[19] David V (2016). Impact of biofilm resuspension on mesozooplankton in a shallow coastal ecosystem characterized by a bare intertidal mudflat. J. Mar. Biol. Assoc. UK.

[20] Chen, X. H. *et al*. Bioturbation as a key driver behind the dominance of Bacteria over Archaea in near-surface sediment. *Sci*. *Rep*. **7** (2017). 10.1038/s41598-017-02295-x PMC5445093 28546547

[21] Saint-Béat B (2104). How does the resuspension of the biofilm alter the functioning of the benthos–pelagos coupled food web of a bare mudflat in Marennes-Oléron Bay (NE Atlantic)?. J. Sea Res..

[22] Abrantes KG, Barnett A, Bouillon S (2014). Stable isotope-based community metrics as a tool to identify patterns in food web structure in east African estuaries. Funct. Ecol..

[23] Zou K, Thébault E, Lacroix G, Barot S (2016). Interactions between the green and brown food web determine ecosystem functioning. Funct. Ecol..

[24] Solan M (2004). *In situ* quantification of bioturbation using time lapse fluorescent sediment profile imaging (f SPI), luminophore tracers and model simulation. Mar. Ecol. Prog. Ser..

[25] Holtmann, S. *et al*., *Atlas of the zoobenthos of the Dutch continental shelf* (Ministry of Transport, Public Works and Water Management Rijswijkwaterstaat, 1996).

[26] Degraer, S. *et al*., *The macrobenthos atlas of the Belgian part of the North Sea* (Belgian Science Policy, 2006).

[27] Queirós A (2013). A bioturbation classification of European marine infaunal invertebrates. Ecol. Evol.

[28] Schiffers, K., Teal, L., Mark, J., Travis, J. & Solan, M., An Open Source simulation model for soil and sediment bioturbation. *Plos One* **6** (2011). 10.1371/journal.pone.0028028 PMC3230619 22162997

[29] Gilbert F (2007). Sediment reworking by marine benthic species from the GullmarFjord (Western Sweden): Importance of faunal biovolume. J. Exp. Mar. Biol. Ecol..

[30] Kleiber M (1932). Body size and metabolism. Hilgardia.

[31] West G, Brown J, Enquist B (1997). A general model for the origin of allometric scaling laws in biology. Science.

[32] Brown, J., Allen, A. & Gillooly, J., In *Body size: the structure and function of aquatic ecosystems* (Cambridge University Press, Cambrige, 2007), pp. 1–15.

[33] Brown J, Gillooly J, Allen A, Savage V, West G (2004). Toward a metabolic theory of Ecology. Ecology.

[34] Brown, J., *Macroecology* (The University of Chicago Press, 1995).

[35] Gaston, K. & Blackburn, T., *Pattern and process in macroecology* (Blackwell Science, 2000).

[36] Marquet P (2002). Of predators, prey, and power laws. Science.

[37] De Roos A, Persson L, McCauley E (2003). The influence of size-dependent life-history traits on the structure and dynamics of populations and communities. Ecol. Lett.

[38] Savage V, Gillooly J, Brown J, West G, Charnov E (2004). Effects of body size and temperature on population growth. Am. Nat..

[39] Yvon-Durocher G (2012). Reconciling the temperature dependence of respiration across timescales and ecosystem types. Nature.

[40] Barneche D (2014). Scaling metabolism from individuals to reef-fish communities at broad spatial scales. Ecol. Letters.

[41] Isaac N, Storch D, Carbone C (2011). Taxonomic variation in size-density relationships challenges the notion of energy equivalence. Biol. Lett.

[42] Allgeier, J., Wengerb, S., Rosemond, A., Schindler, D. & Layman, C., Metabolic theory and taxonomic identity predict nutrient recycling in a diverse food web. *PNAS* **112** (2015). 10.1073/pnas.1420819112 PMC4443305 25877152

[43] Barneche R, Allen A (2015). Embracing general theory and taxon-level idiosyncrasies to explain nutrient recycling. PNAS.

[44] Clark R, Zera A, Behmer T (2016). Metabolic rate is canalized in the face of variable life history and nutritional environment. Funct. Ecol..

[45] Shumway S (1979). The effects of body size, oxygen tension and mode of life on the oxygen uptake rates of polychaetes. Comp. Biochem. Phys. A.

[46] Cammen L (1985). Metabolic loss of organic carbon by the polychaete Capitella capitata (Fabricius) estimated from initial weight decrease during starvation, oxygen uptake, and release of 14 C by uniformly-labeled animals. Mar. Ecol. Prog. Ser.

[47] Vladimirova I, Kleimenov S, Radzinskaya L (2003). The relation of energy metabolism and body weight in bivalves (Mollusca: Bivalvia). Biology Bull.

[48] Cadee G (1976). Sediment reworking by Arenicola marina on tidal flats in the Dutch Wadden Sea. Neth. J. Sea Res..

[49] Cammen L (1980). Ingestion rate - empirical model for aquatic deposit feeders and detritivores. Oecologia.

[50] Smaal A, Vonck A, Bakker M (1997). Seasonal variation in physiological energetics of Mytilus edulis and Cerastoderma edule of different size classes. J. Mar. Biol. Assoc. UK.

[51] Compton TJ (2106). Burrowing behavior of a deposit feeding bivalve predicts change in intertidal ecosystem state. Front. Ecol. Evol.

[52] Damuth J (1981). Population density and body size in mammals. Nature.

[53] Damuth J (1991). Ecology - Of size and abundance. Nature.

[54] Schmid P, Tokeshi M, Schmid-Araya J (2002). Scaling in stream communities. P. Roy. Soc. Lond. B Bio.

[55] Allen A, Gillooly J, Brown J (2005). Linking the global carbon cycle to individual metabolism. Funct Ecol..

[56] Mehta, A. & Partheniades, E., *Resuspension of deposited cohesive sediment beds*, presented at 18th Conference on Coastal Engineering, (unpublished) 1982.

[57] van Prooijen, B. & Winterwerp, J., A stochastic formulation for erosion of cohesive sediments. *J*. *Geophys*. *Res*. **115** (C01005).

[58] Lee, H. & Swartz., R. C., In *Contaminants and sediment*, edited by Baker, R. (Environmental Protection Agency, 1980).

[59] Clark A, Johnston N (1999). Scaling of metabolic rate with body mass and temperature in teleost fish. J. Anim. Ecol..

[60] Maire O, Duchene J, Rosenberg R, de Mendonca J, Gremare A (2006). Effects of food availability on sediment reworking in Abra ovata and A. nitida. Mar. Ecol. Prog. Ser..

[61] Widdows J, Brinsley M, Salkeld P, Elliott M (1998). Use of annular flumes to determine the influence of current velocity and bivalves on material flux at the sediment-water interface. Estuaries.

[62] Brey T (2010). An empirical model for estimating aquatic invertebrate respiration. Methods Ecol. Evol.

[63] Zwarts L, Blomert A, Spaak P, de Vries B (1994). Feeding radius, burying depth and siphon size of Macoma balthica and Scrobicularia plana. J. Exp. Mar. Biol. Ecol..

[64] Cozzoli F, Bouma T, Ysebaert T, Herman P (2013). Application of non-linear quantile regression to macrozoobenthic species distribution modelling: comparing two contrasting basins. Mar. Ecol. Prog. Ser..

[65] Volkenborn N, Robertson D, Reise K (2009). Sediment destabilizing and stabilizing bio-engineers on tidal flats: cascading effects of experimental exclusion. Helgoland Mar. Res..

[66] DeLong JP, Hanley TC, Vasseur DA (2014). Competition and the density dependence of metabolic rates. J. Anim. Ecol..

[67] Nadler LE, Killen SS, McClure EC, Munday PL, McCormick MI (2016). Shoaling reduces metabolic rate in a gregarious coral reef fish species. J. Exp. Biol..

[68] Gjoni, V., Cozzoli, F., Rosati, I. & Basset, A., Size–Density Relationships: a Cross-Community approach to benthic macroinvertebrates in Mediterranean and Black Sea Lagoons. *Estuar*. *Coast*. **40** (4) (2016).

[69] Mouillot D (2006). Alternatives to taxonomic-based approaches to assess changes in transitional water communities. Aquat. Conserv..

[70] Gillooly J, Charnov E, West G, Savage M, Brown J (2001). Effects of size and temperature on developmental time. Nature.

[71] Ernest S (2003). Thermodynamic and metabolic effects on the scaling of production and population energy use. Ecol. Lett.

[72] Gillooly J, Allen A, Savage V, Charnov E, West G (2006). Response to Clarke and Fraser: effects of temperature on metabolic rate. Funct. Ecol..

[73] Clarck A (2006). Temperature and the metabolic theory of ecology. Funct. Ecol..

[74] Pörtner H, Farrell P (2008). Physiology and Climate Change. Science.

[75] Kristensen E (1983). Ventilation and oxygen uptake by 3 species of Nereis (Annelida: Polychaeta). II. Effects of temperature and salinity changes. Mar. Ecol. Prog. Ser..

[76] Ouellette D (2004). Effects of temperature on *in vitro* sediment reworking processes by a gallery biodiffusor, the polychaete Ne. Mar. Ecol. Prog. Ser..

[77] Baranov V, Lewandowski J, Krause S (2016). Bioturbation enhances the aerobic respiration of lake sediments in warming lakes. Biol. Letters.

[78] Sheppard C (2004). Sea surface temperature 1871–2099 in 14 cells around the United Kingdom. Mar. Poll. Bull.

[79] Sommer A, Pörtner H (2002). Metabolic cold adaptation in the lugworm Arenicola marina: Comparison of a North Sea and a White Sea population. Mar. Ecol. Prog. Ser..

[80] Irie T, Fischer K (1999). Ectotherms with a calcareous exoskeleton follow the temperature-size rule-evidence from field survey. Mar. Ecol. Prog. Ser..

[81] Daufresne M, Lengfellner K, Sommer U (2009). Global warming benefits the small in aquatic ecosystems. PNAS.

[82] Ysebaert T, Herman P (2002). Spatial and temporal variation in benthic macrofauna and relationships with environmental variables in an estuarine, intertidal soft-sediment environment. Mar. Ecol. Prog. Ser..

[83] Rysgaard S, Christensen P, Nielsen L (1995). Seasonal variation in nitrification and denitrification in estuarine sediment colonized by benthic microalgae and bioturbating infauna. Mar. Ecol. Prog. Ser..

[84] Teal L, M. B, Parker E, Solan M (2008). Global patterns of bioturbation intensity and mixed depth of marine soft sediments. Aquat. Biol.

[85] Cozzoli F (2014). A mixed modeling approach to predict the effect of environmental modification on species distributions. Plos One.

[86] Cozzoli F (2017). A modeling approach to assess coastal management effects on benthic habitat quality: A case study on coastal defense and navigability. Estuar. Coast. Shelf S.

[87] Ellis J (2017). Multiple stressor effects on marine infauna: responses of estuarine taxa and functional traits to sedimentation, nutrient and metal loading. Sci. Rep.

[88] Harris J (2015). Biotic interactions influence sediment erodibility on wave-exposed sandflats. Mar. Ecol. Prog. Ser..

[89] Kristensen E (2013). Influence of benthic macroinvertebrates on the erodability of estuarine cohesive sediments: Density- and biomass-specific responses. Estuar. Coast. Shelf S.

[90] Lindström Swanberg I (1991). The influence of the filter-feeding bivalve L. on microphytobenthos: a laboratory study. J. Exp. Mar. Biol. Ecol..

[91] Colijn F, Dijkema K (1981). Species composition of benthic diatoms and distribution of Chlorophyll a on an intertidal flat in the Dutch Wadden Sea. Mar. Ecol. Prog. Ser..

[92] Vos, P., Misdorp, R. & De Boer, P. In Tide-Influenced Sedimentary Environments and Facies, edited by De Boer, P. L., Van Gelder, A. & Nio, S. D. (Reidel, 1998), pp. 511–526.

[93] Sutherland T, Grant J (1998). The effect of carbohydrate production by the diatom Nitzschia curvilineata on the erodibility of sediment. Limnol. Ocenogr.

[94] Tolhurst T (2006). Small-scale temporal and spatial variability in the erosion threshold and properties of cohesive intertidal sediments. Cont. Shelf Res..

[95] Montserrat F, Van Colen C, Degraer S, Ysebaert T, Herman PMJ (2008). Benthic community-mediated sediment dynamics. Mar. Ecol. Prog. Ser..

[96] Rakotomalala C, Grangeré K, Ubertini M, Forêt M, Orvain F (2015). Modelling the effect of Cerastoderma edule bioturbation on microphytobenthos resuspension towards the planktonic food web of estuarine ecosystem. Ecol. Model..

[97] Sousa T, Domingos T, Kooijman SALM (2008). From empirical patterns to theory: a formal metabolic theory of life. Philos. T. R. Soc. of Lond. B.

[98] Williamson C, Grad G, De Lange H, Gilroy S, Karapelou D (2002). Temperature-dependent ultraviolet responses in zooplankton: Implications of climate changes. Limnol. Oceanogr..

[99] Flach E (1996). The influence of the cockle, Cerastoderma edule, on the macrozoobenthic community of tidal flats in the Wadden Sea. Mar. Ecol. - PSZNI.

[100] Hughes R (1969). A study of feeding in Scrobicularia plana. J. Mar. Biol. Assoc. UK.

[101] Purchon, R. D., *The Biology of Mollusca − 2nd Edition* (Pergamon Press Ltd., 1997).

[102] Yonge C (1949). On the structure and adaptations of the Tellinacea, deposit-feeding Eulamellibranchia. Philos. T. R. Soc. Lon. B.

[103] Brey, T., Population dynamics in benthic invertebrates, Available at http://www.thomas-brey.de/science/ (2001).

[104] R-Core-Team, R: A language and environment for statistical computing. R Foundation for Statistical Computing, Vienna, Austria, Available at www.R-project.org (2013).

