# Supplementary material for: The combined influence of body size and density on cohesive sediment resuspension by bioturbators
Source: Sci Rep. 2018 Mar 1;8:3831. doi: 10.1038/s41598-018-22190-3 (PMC5832813; doi:10.1038/s41598-018-22190-3)
Supplement: Supplementary file 1 — Supplementary information [file 41598_2018_22190_MOESM1_ESM.pdf]

The combined influence of body size and density on cohesive sediment resuspension by bioturbators

Appendix A – Estimation of individual and community metabolic rates

Appendix B – Experimental flume, schemes and calibration

Appendix C – Additional data analysis

Francesco Cozzoli[*†a], Tjeerd J. Bouma[a], Pauline Ottolander[a], Maria Salvador Lluch[a], Tom Ysebaert[a,b] and Peter M. J. Herman[a]

[a] Department of Estuarine and Delta Systems, Royal Netherlands Institute of Sea Research (NIOZ) and Utrecht University, Yerseke, the Netherlands

[b] Institute for Marine Resources and Ecosystem Studies (IMARES), Wageningen University, Wageningen, the Netherlands

[*] Corresponding author: francesco.cozzoli@unisalento.it

[†]Mailing address: Dipartimento di Scienze e Tecnologie Biologiche ed Ambientali, Centro Ecotekne Pal. B S.P. 6, Lecce - Monteroni - Lecce – Italy

Running Headline: Size and density allometry of bioturbators

Appendix A – Estimation of individual and community metabolic rates

Estimation of individual body mass

The bioturbators' body sizes were selected in a way to cover the natural range of each analysed species (*e.g.* 1, 2, 3, 4) and according to the availability of experimental organisms (Table A1). For bivalves, shell length was measured by a calliper in integer mm and only individuals of exact length were selected. Conversion from individual length to ash free dry weight (g AFDW) was performed according to the scaling relationships provided from the NIOZ – Yerseke Monitor Taskforce (Table A2). Measuring the size of live *A. marina* is complex due to the variable length of living specimens. In this case, we proceed prior to experiment by starving the specimens for 2 days in clean marine waters to allow them to expel the sediment in their gut. *A. marina* specimens were visually selected according to their size, cleaned from mucus and sediment by gently rolling them on absorbent paper, weighed and assigned to groups of approximately equal body mass. For the two smaller size classes of *A. marina* we were able to select individuals with a wet weight within +/- 5% of the class modal value (160 and 1500 mg wet weight). For the largest size class of *A. marina* we were able to select individuals with a wet weight within +/- 2.5% of the class modal value (8000 mg wet weight). Conversion between *A. marina* wet weight and AFDW were performed according to the scaling relationship provided from the NIOZ – Yerseke Monitor Taskforce (Table A2).

Estimation of individual metabolic rates

Bioturbators' individual metabolic rates were estimated according to the empirical model for acquatic macroinvertebrates respiration of Brey (5) using a trait classification for sessile intertidal satiate Anellida and Bivalvia Heterodonta at temperature of 18 ºC. We assumed an average energy density of 21.5 J mg^{-1} (6). Metabolic rate and 95% Confidence Intervals were estimates using the spreadsheet available at:

http://www.thomas-brey.de/science/virtualhandbook/spreadsheets/index.html.

This spreadsheet easily allows estimating individual metabolic rates according to size, temperature, water depth and information on specimen taxonomy and lifestyle.

In this work, we used the empirical model of Brey (5) to have a more accurate estimate of individual metabolic rate and confidence interval. However, the generic model for metabolic rates scaling $I=aM^{0.75}e^{-E/kT}$ may also provide a good and more direct approximation of individual metabolic rate (Figure A1, red dashed line). It follows that the population or community metabolic rate can be approximated as $I_{TOT} = (aM_{av}^{0.75}e^{-E/kT})$*N where M_{av} and N are respectively the average individual size and the individuals density.

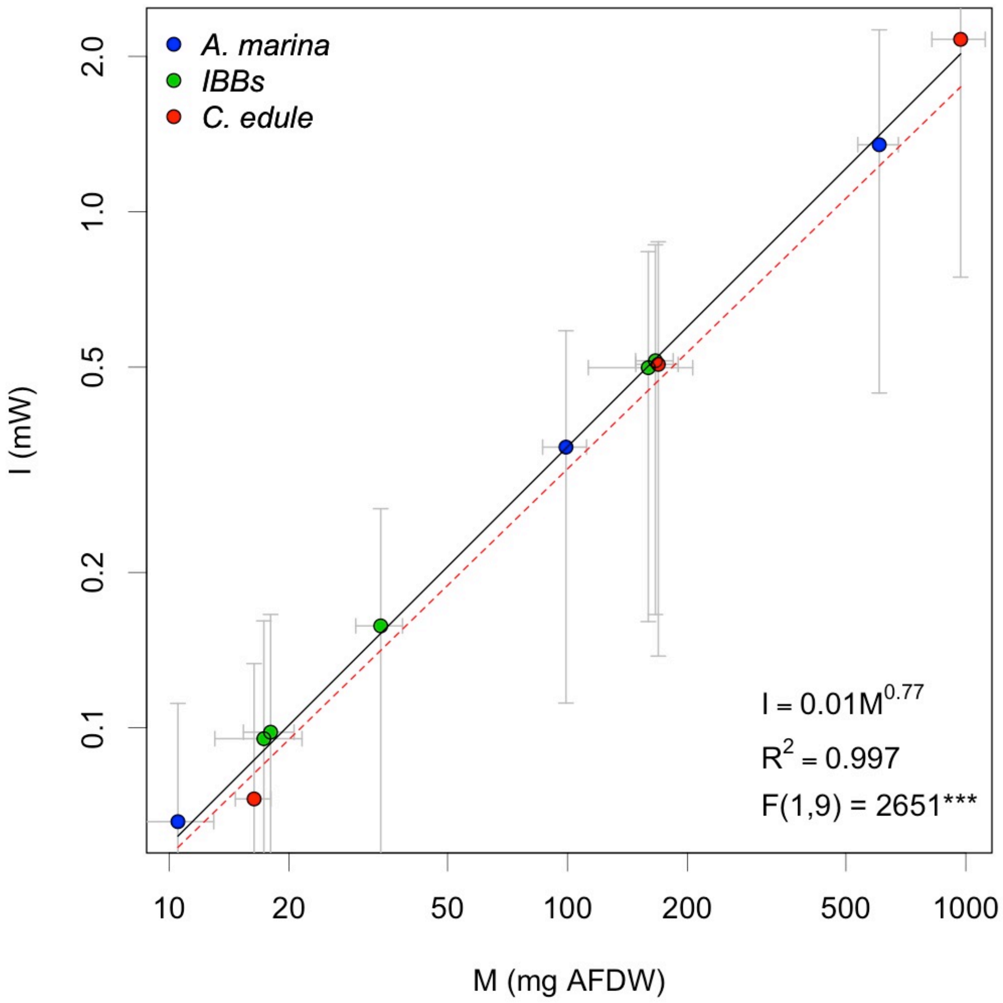

Figure A1: Scaling relationship between individual body mass M (mg AFDW) and individual metabolic rate I (mW) as predicted from to the the empirical model of Brey (5) for deep (*A. marina*, red) intermediate (*IBBs*, green) and shallow (*C. edule*, blue) bioturbators. The horizontal grey error bars show the 95% CI on estimated individual AFDW related to errors in specimen measurements and conversion between shell length or wet weight and AFDW. The vertical grey error bars show the 95CI related to unexplained variance in the Brey's model. The full black line shows the predicted mass-metabolic rate scaling trend. The dashed red line shows the scaling trend expected from energetic theories ($I=aM^{0.75}$)

Table A1: Selected bioturbators sizes. Specimens were selected according to their shell length (mm, bivalves) or wet weight (mg, *A. marina*).

**Species**	**Size**	
	mm/mg	+/- Error
A. alba	15	0.5
A. marina	160	8
A. marina	1500	75
A. marina	8000	200
C. edule	35	0.5
C. edule	20	0.5
C. edule	10	0.5
L. balthica	15	0.5
S. plana	15	0.5
S. plana	35	0.5
R. philippinarum	25	0.5

Table A2: Relationships between individual body length (bivalves) or wet weight (*A. marina*) and individual AFDW were estimated as power laws ($Y=aX^b$) from data collected in the Westerschelde and Oosterschelde between 2011 and 2013 and provided from the NIOZ – Yerseke Monitor Taskforce. For each relationship we reported the estimated coefficients (log(a), b) +/- 95% Confidence Interval, number of observations (N), explained variance (R^2) and the size range on which the relationship was calculated.

**Species**	**log(a)**	**+/-95CI**	**b**	**+/-95CI**	**N**	**R^2**	**Min. Size**	**Max. Size**
A. alba[1]	-5.26	0.31	3	0.13	66	0.89	4	19
C. edule[1]	-5.1	0.13	3.24	0.04	175	0.97	5	43
L. balthica[1]	-4.64	0.09	3.02	0.03	542	0.93	3	23
S. plana[1]	-4.22	0.15	2.63	0.05	146	0.95	5	47
R. philippinarum[1]	-5.06	0.35	3.15	0.12	13	0.98	5	45
A. marina[2]	-2.5	0.11	1.04	0.02	186	0.94	32.8	4292.5

[1] *length (mm) to mass (mg AFDW) conversion*
[2] *mass (mg wet weight) to mass (mg AFDW) conversion*

Appendix B – Experimental flume, schemes and calibration

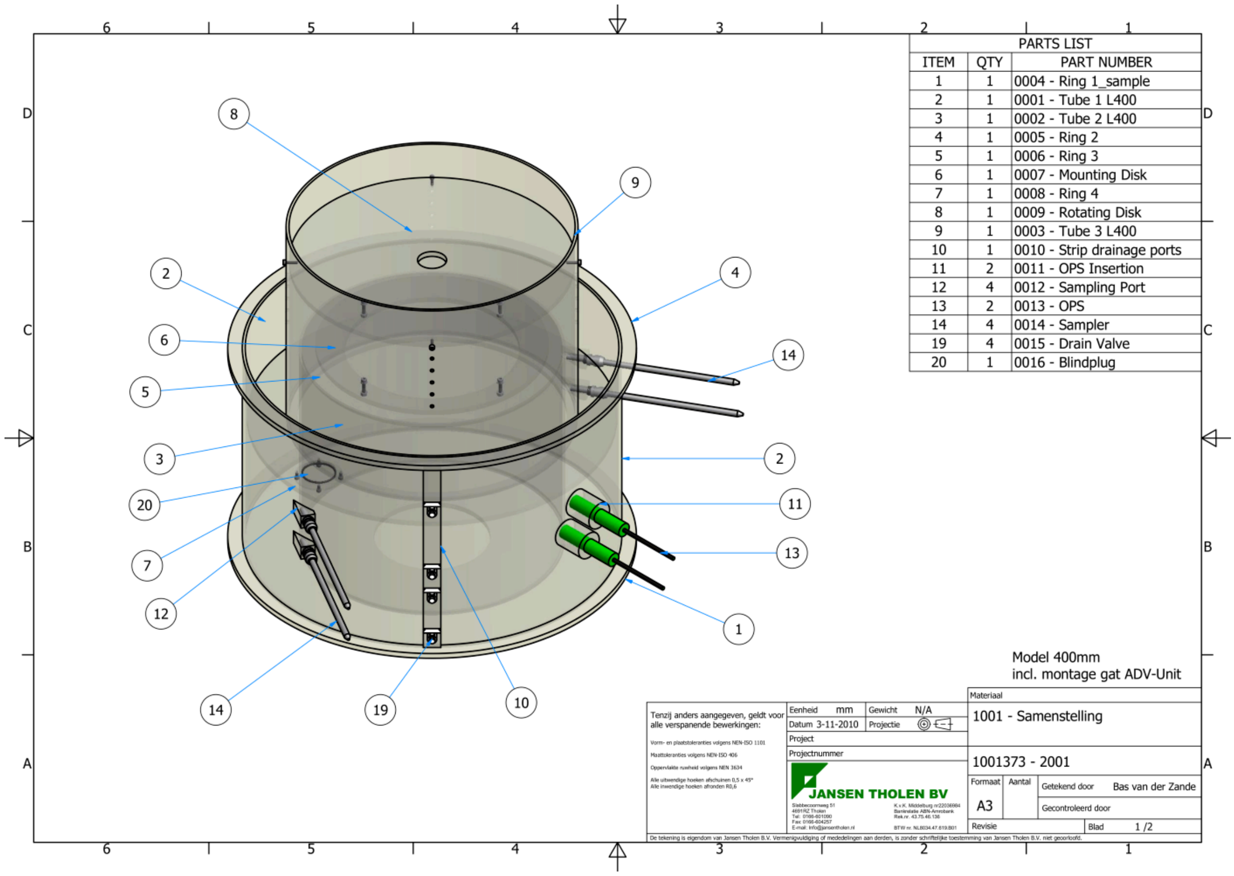

Figure B1: Schematic diagram of the annular flumes used in this work (40 cm height model). Technical drawing and flumes realization: Jansen Tholen B.V. (http://www.jansentholen.nl/)

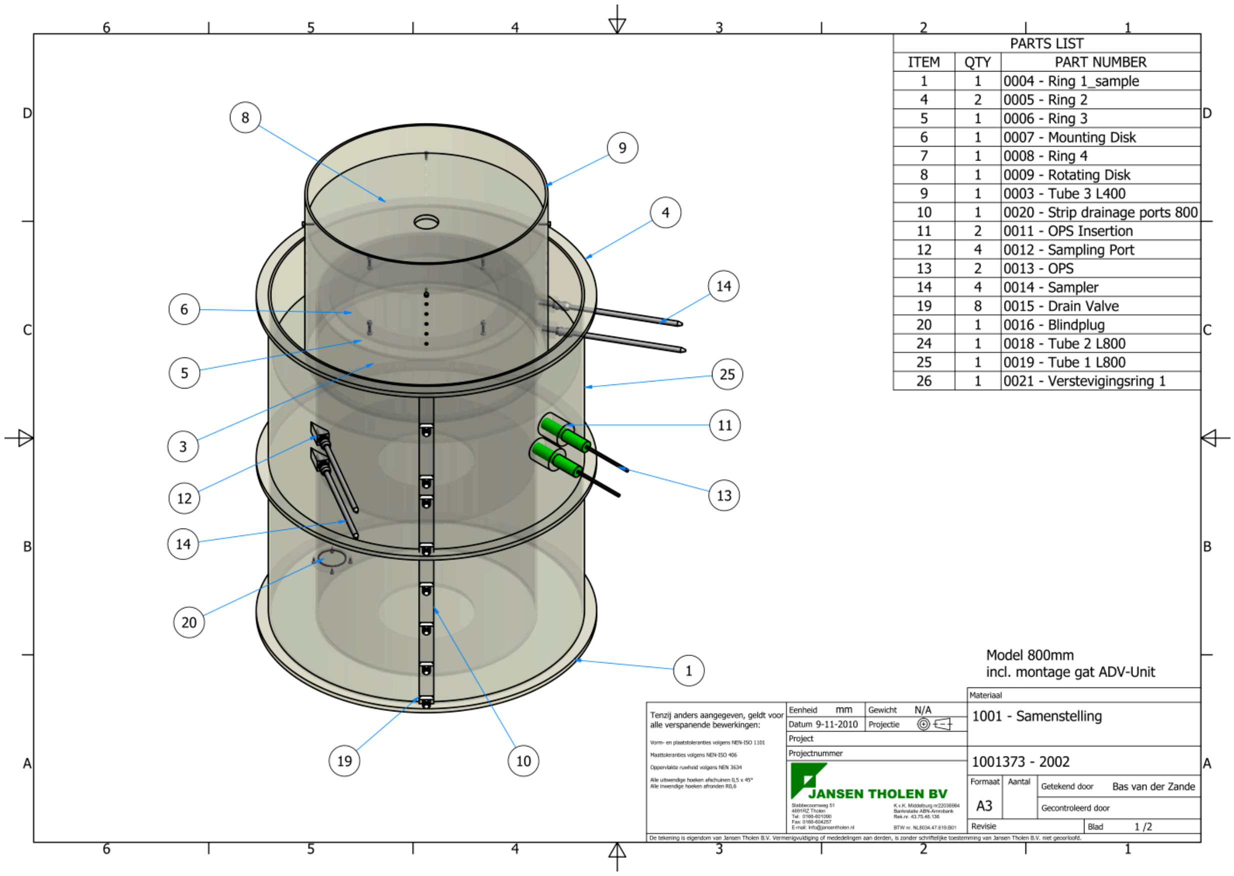

Figure B2: Schematic diagram of the annular flumes used in this work (80 cm height model). This flume was used for the larger sizes of *A. marina*. Technical drawing and flume realization: Jansen Tholen B.V. (http://www.jansentholen.nl/)

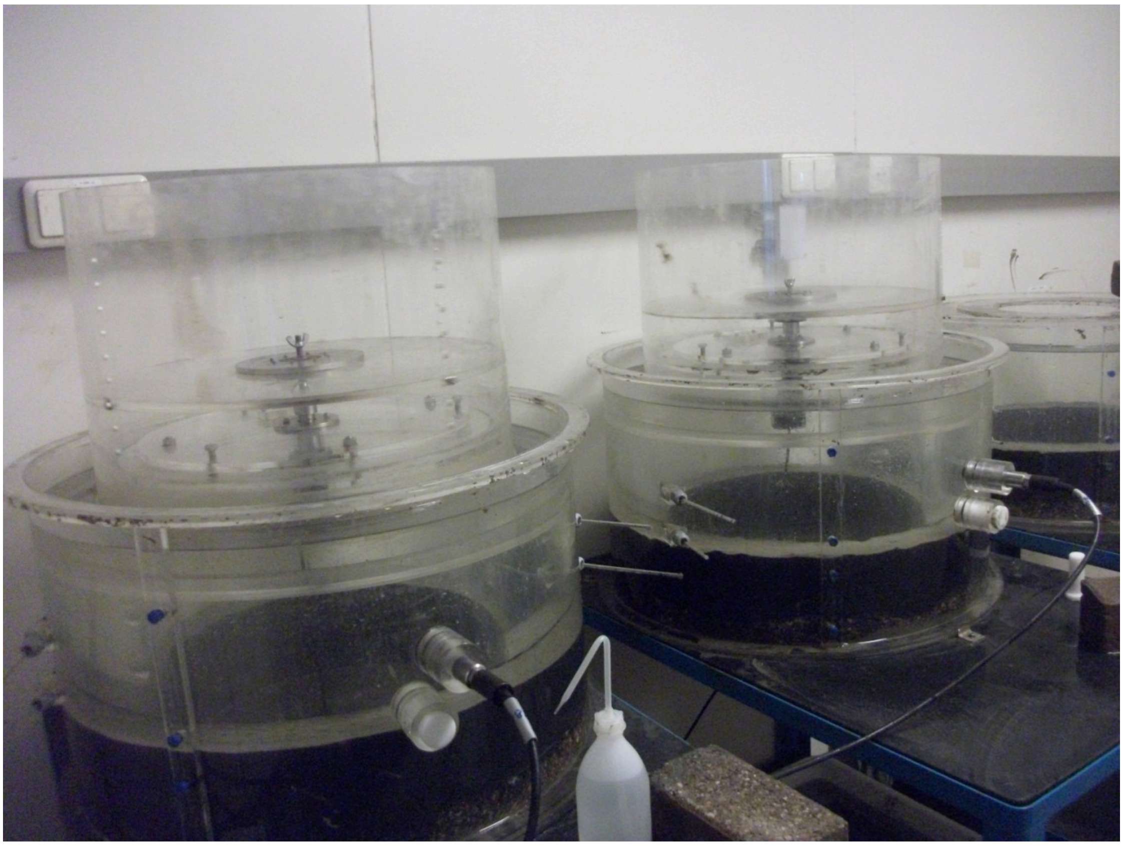

Figure B3: Running experiments. Experimental animals were buried in the sediment matrix (median grain size = 100 µm, silt content 12%). Water motion (current velocity of 30 cm sec^{-1}) was generated by rotating discs at the top of the flumes. Water turbidity (as a proxy of the amount of resuspended sediment) was measured by the optical backscatter sensors (OBS 3+, Campbell scientific) inserted into the lateral sampling ports.

Table B1: Water turbidity (Nephelometric Turbidity Unit, NTU), as a proxy of resuspended sediment, was measured using an optical backscatter sensor and converted into Suspended Sediment Concentration (SSC, g L^{-1}) according to the exponential relationship SSC ~ e^{a+bNTU}. Water samples for calibration were collected at the same height at which the OBS3+ sensors were placed to avoid bias related to sediment stratification in the water column.

.

	Est.	*95% CI*	*P*
a	-2.06	-3.00 – -1.12	<.001
b	0.01	0.01 – 0.02	<.001
Observations		11	
R^2 / adj. R^2		.745 / .717	
F-statistics		26.323***	

Appendix C – Additional data analysis

Table C1: Summary table of the ANOVA CRS~Species, considering only species belonging to the group Intermediate Burrowers Bivalves for which similar sizes and densities were tested (Table C2)

	R_{TOT} ~ *Species*		
	Est.	*95% CI*	*p*
Intercept	36.81	26.56 – 47.07	<.001
L. balthica	-1.63	-14.19 – 10.93	.762
S. plana	8.15	-6.36 – 22.66	.218
R. philippinarum	-2.82	-17.32 – 11.69	.652
Observations	10		
R^2 / adj. R^2	.428 / .142		
F-statistics	1.495		

Table C2: List of runs included in the previous ANOVA analysis (Table B1). Only runs for the "Intermediate Burrowers Bivalves' category with similar numbers of individuals and sizes were tested

	Size		M		I		N	I_{TOT}		R_{TOT}
Species	mg/mm	±95CI	mg AFDW	±95CI	mW	±95CI	N of Ind. m^{-2}	mW m^{-2}	±95CI	g m^{-2}
A. alba	15	0,5	17,29	17,29	0,10	0,03	45		4,25	32,64
A. alba	15	0,5	17,29	17,29	0,10	0,03	45		4,25	40,99
L. balthica	15	0,5	33,98	33,98	0,16	0,04	32		5,02	32,75
L. balthica	15	0,5	33,98	33,98	0,16	0,04	32		5,02	35,73
L. balthica	15	0,5	33,98	33,98	0,16	0,04	64		10,03	33,49
L. balthica	15	0,5	33,98	33,98	0,16	0,04	64		10,03	38,77
S. plana	15	0,5	17,98	17,98	0,10	0,02	64		6,24	40,26
S. plana	15	0,5	17,98	17,98	0,10	0,02	64		6,24	49,67
R. philippinarum	25	0,5	159,54	159,54	0,50	0,17	32		15,87	26,58
R. philippinarum	25	0,5	159,54	159,54	0,50	0,17	32		15,87	41,41

Table C3: Summary of the regression models between R_{TOT} (g m^{-2}) and bioturbators population metabolic power (I_{TOT}, mW m^{-2}) excluding the two observations with higher I_{TOT} and leverage on the regression model.

	Est.	*95% CI*	*p*
c	39.87	33.56 – 46.18	<.001
I_{TOT}	0.17	0.03 – 0.32	.017
Observations		30	
R^2 / adj. R^2		.186 / .157	
F-statistics		6.418*	

Works Cited

1. Cadee, G., Sediment reworking by Arenicola marina on tidal flats in the Dutch Wadden Sea. *Netherlands J. Sea Res.* **10**, 440-460 (1976).
2. Holtmann, S. *et al.*, *Atlas of the zoobenthos of the Dutch continental shelf* (Ministry of Transport, Public Works and Water Management Rijswijkwaterstaat, 1996).
3. Zebe, E. & Schiedek, D., The lugworm Arenicola marina: A model of physiological adaptation to life in intertidal sediments. *Helgolander Meersun* **50**, 37–68 (1996).
4. Degraer, S. *et al.*, *The macrobenthos atlas of the Belgian part of the North Sea* (Belgian Science Policy, 2006).
5. Brey, T., An empirical model for estimating aquatic invertebrate respiration. *Met. Ecol. Evol.* **1**, 92–101 (2010).
6. Brey, T., Population dynamics in benthic invertebrates, Available at HYPERLINK "http://www.thomas-brey.de/science/" http://www.thomas-brey.de/science/ (2001).
